# Inverse Transformation in Eddy Current Tomography with Continuous Optimization of Reference Defect Parameters

**DOI:** 10.3390/ma14174778

**Published:** 2021-08-24

**Authors:** Paweł Nowak, Roman Szewczyk, Anna Ostaszewska-Liżewska

**Affiliations:** 1 Institute of Metrology and Biomedical Engineering, Warsaw University of Technology, 02-495 Warsaw, Poland; anna.ostaszewska@pw.edu.pl; 2 Łukasiewicz Research Network—Industrial Research Institute for Automation and Measurements PIAP, 02-486 Warsaw, Poland; rszewczyk@piap.pl

**Keywords:** inverse tomography transformation, eddy current tomography, finite element method

## Abstract

This paper presents a methodology of inverse tomography transformation in eddy current tomography with the use of continuous optimization of reference defect parameters. Ferromagnetic steel samples with rectangular air inclusion defects of known dimensions were prepared and measured using an eddy current tomography setup. FEM-based (Finite Element Method based) forward tomography transformation was developed and utilized in inverse tomography transformation. The presented method of inverse tomography transformation is based on the continuous optimization of parameters that can describe the sample, such as the diameter and dimensions of the reference defect. The obtained results of inverse tomography transformation were in high accordance with the real parameters of the samples. Additionally, the presented method had acceptable repeatability. The obtained values of the sample parameters fit within the range of expanded uncertainty when compared to the real parameters of the sample.

## 1. Introduction

Eddy current tomography (or magnetic induction tomography) is a non-destructive, contactless method for evaluating discontinuities in a conductive material [1]. It is based on the standard eddy current non-destructive evaluation method [2,3,4,5], where the tested object influences the coupling of at least two coils. ECT (eddy current tomography) setups either collect data from multiple sets of coils [6,7,8,9] or conduct measurements for different positions of the sample [10,11].

A typically measured signal in ECT is the voltage induced in the coil, which is caused by an alternating magnetic field. The parameters of the measurand are the signal amplitude and phase shift with respect to the excitation signal. The variation of these parameters is an implicit function, the value of which varies on the signal frequency, the electromagnetic properties of the tested material, and its spatial distribution in the tomograph region of interest.

Eddy current tomography has many applications, such as magnetic spectroscopy [9,12] (determining the permeability of a material as a function of measurement frequency), assessing phase changes in matter [13,14], or even assessing changes in the state of biological tissues [15,16]. In this paper, a method for the utilization of ECT in the non-destructive evaluation of objects is presented. We focus on the shape reconstruction of an axisymmetric object with a possible defect. The presented method can be utilized in quality assurance systems, in which it will provide binary information about object suitability. Additionally, it can provide information about the location and dimensions of cracks. This information can be used for proper trimming of production lines or locating failing machine tools.

In ECT, based on the measurement data, the spatial distribution of the material can be obtained in the process of inverse tomography transformation [17]. The procedure for the development of inverse tomography transformation is complicated due to the nature of the phenomenon of eddy current induction. The effect is described by ill-posed differential equations and cannot be solved analytically except in a few simple cases. Thus, a more universal reconstruction requires the use of a numerical method, such as the finite element method, to approximate the solution of eddy current induction and its influence on the spatial distribution of the magnetic field.

Previous research in the field of inverse tomography transformation has focused on the total reconstruction of the shape of the tested object based on pre-calculated sensitivity maps of the tomography setup [18] or with the use of simplified two-dimensional modeling [14]. Both methods were created due to the significant computational cost of three-dimensional modeling of magnetic induction distribution.

In this paper, we present a method of inverse eddy current tomography suitable for non-destructive testing application with three-dimensional finite element modeling. This allows for the proper reconstruction of the phenomenon of eddy current induction in a sample and calculates its influence on the magnetic field distribution in the tomography setup.

## 2. Materials and Methods

### 2.1. Sample Preparation

**Figure 1:**
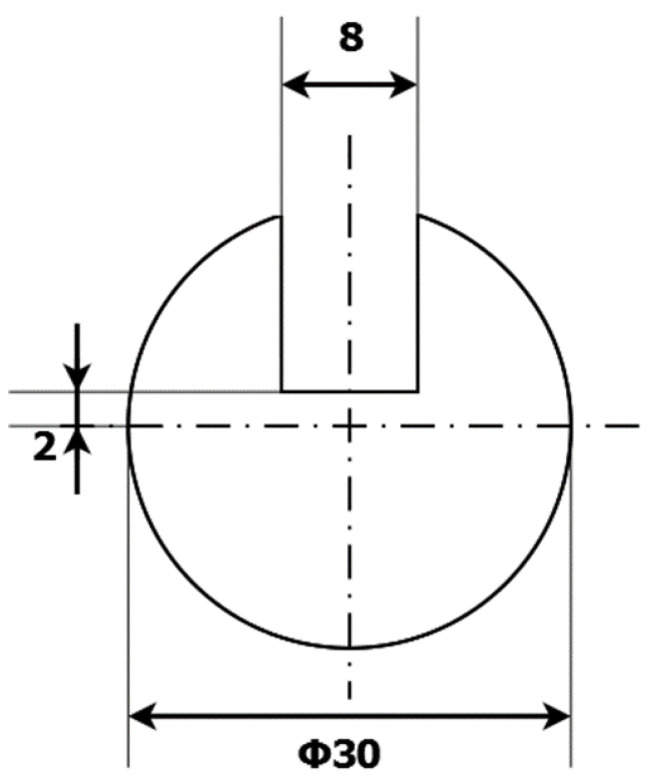
Cross-section of the sample showing a reference defect with a width of 8 mm.

For this research, cylindrical samples with a reference defect were prepared. The samples were made of S235JR steel, (Łukasiewicz Research Network—Industrial Research Institute for Automation and Measurements PIAP, Warsaw, Poland) which is one of the most used materials for welded constructions, load-bearing structures, and dynamically loaded structures. The samples were in the form of a cylinder with a 30 mm diameter and a 120 mm height. The defect was a rectangular indentation with a constant depth of 13 mm and varying widths of 2, 4, 6, 8, 10, and 12 mm. The cross-section of the exemplary sample with an 8 mm wide defect is presented in Figure 1.

### 2.2. Measurement Method

**Figure 2:**
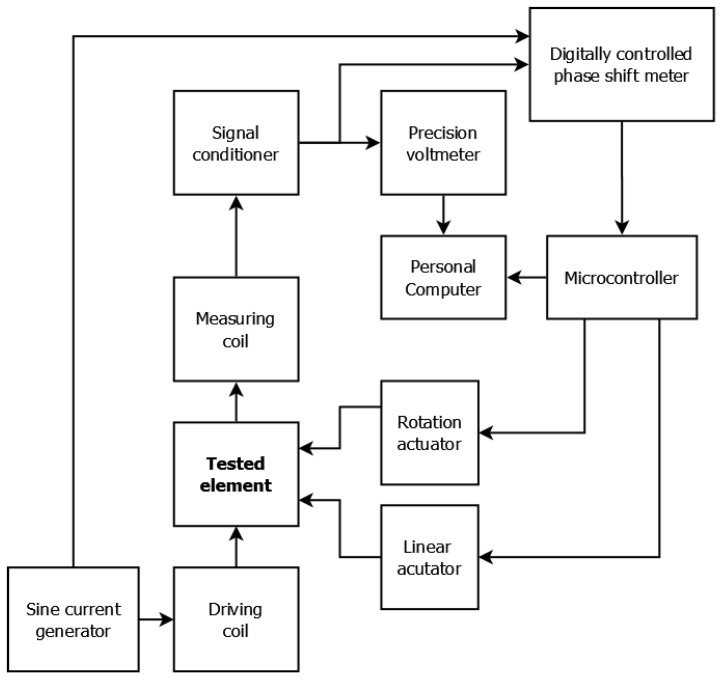
Block diagram of the utilized eddy current tomography setup.

The samples were measured on an eddy current tomography setup (Figure 2) [10]. The tested object was placed on the rotary actuator (Wobit, Pniewy, Poland), which was placed on the linear actuator (Wobit, Pniewy, Poland). The tested sample and rotation actuator were moved perpendicularly to the coil axis, and for each linear step, the sample rotated around its axis in 100 discrete steps. The position of the sample was set by two stepper motors, controlled by an ARM 1114 microcontroller (NXP Semiconductors, Eindhoven, The Netherlands).

The sample was transferred between two air–core co-axial coils (driving and measuring). The measuring coil had a 6.1 mm internal radius and a 14.8 mm external radius, whereas the excitation coil had a 7.4 mm internal radius and a 17.9 mm external radius. Both coils consisted of 100 turns. An alternating current of 2 kHz powered the driving coil, which induced an alternating magnetic field that caused the induction of eddy currents in the conducting sample. The shape of the eddy current loop strongly depends on the distribution of electromagnetic parameters in the sample’s material, as well as on the object’s geometry, and is very sensitive to any discontinuities. The magnetic field of the measuring coil is a superposition of the initial magnetic field and the magnetic field induced by eddy current. The changes caused by the presence of the sample concern both the amplitude of the measured field and the phase shift between the excitation signal and the measured signal. The phase shift was measured by a digital phase shift meter, and the amplitude signal was measured by a 6½-digit AC voltmeter (TH1961, Tonghui, China). The entire measurement process was controlled by software developed in LabVIEW (National Instruments, Austin, TX, USA).

**Figure 3:**
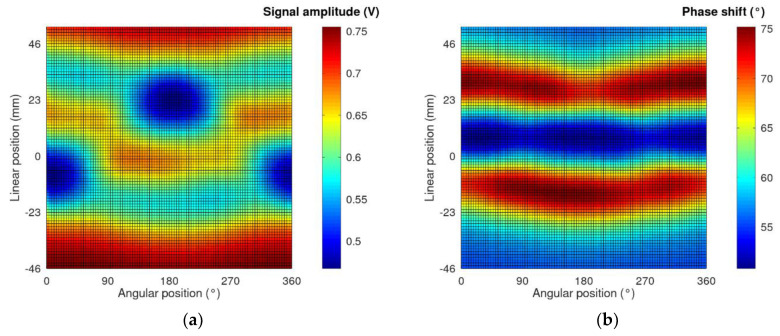
Exemplary measurement results for the sample with an 8 mm wide insertion cut. (**a**) Results of the signal amplitude as a function of the sample position; (**b**) results of the phase shift between the excitation and measured signals as a function of the sample position.

As a result of the measurement in the eddy current tomography setup, matrices representing the measurement signal amplitude and phase shift for each linear and angular position were obtained. Exemplary measurement results are presented in Figure 3. The values on the *x*-axis are angular positions of the sample, which are set by the rotation actuator. The values on the *y*-axis are changes in the linear position with respect to the center of the eddy current tomography setup.

In the case of ferromagnetic and electrically conducting samples (such as those presented, made of S235JR steel), the change in the measurement signal amplitude is caused by the following phenomena. Firstly, it is influenced by the increasing presence of a high-permeability object in the tomograph area with each linear step. This increases the signal amplitude, as presented previously in [19]. Secondly, the induction of eddy currents in the material lowers the amplitude, according to Lenz’s law. The resulting change is a superposition of effects caused by these phenomena.

### 2.3. Method of Inverse Tomography Transformation

Inverse tomography transformation is used to reconstruct the properties of the measured object. Due to the fact that the phenomenon of eddy current induction is described by ill-posed differential equations, analytical inversion is impossible. The proposed method of inverse tomography transformation requires the utilization of FEM modeling and an optimization algorithm. The objective function for the optimization algorithm is to minimize the mean square difference between the measurement results and FEM-based forward tomography transformation, as presented in (1):

(1) $$Q=\frac{\sum_{w=1}^{n}\sum_{k=1}^{m}({\left(T_{Aw,k}-P_{Aw,k}\right)}^{2}+{\left(T_{Pw,k}-P_{Pw,k}\right)}^{2})}{n\cdot m}$$

where:

*Q*—value of the objective function for the optimization algorithm (fit quality factor);

*n*—number of linear positions of the sample;

*m*—number of angular positions of the sample for each linear position;

*T_Aw,k_*—normalized value of the signal amplitude obtained by forward tomography transformation for an element in position (*w,k*);

*P_Aw,k_*—normalized value of the signal amplitude obtained during measurements for an element in position (*w,k*);

*T_Fw,k_*—normalized value of the phase shift obtained by forward tomography transformation for an element in position (*w,k*);

*P_Fw,k_*—normalized value of the signal phase shift obtained during measurements for an element in position (*w,k*).

**Figure 4:**
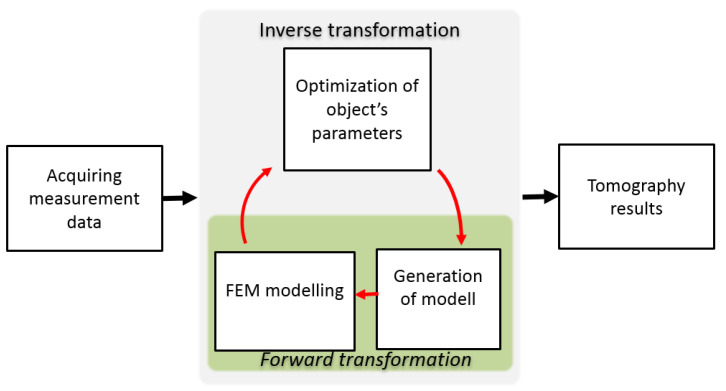
General scheme of inverse tomography transformation for eddy current tomography [20].

A diagram of the utilized method for inverse tomography transformation is presented in Figure 4 [20]. Inverse transformation processes are as follows. Initially, the measurement data are acquired. These data are compared with the results of FEM-based forward tomography transformation for the initial model, and the value of the objective function is calculated by (1).

**Figure 5:**
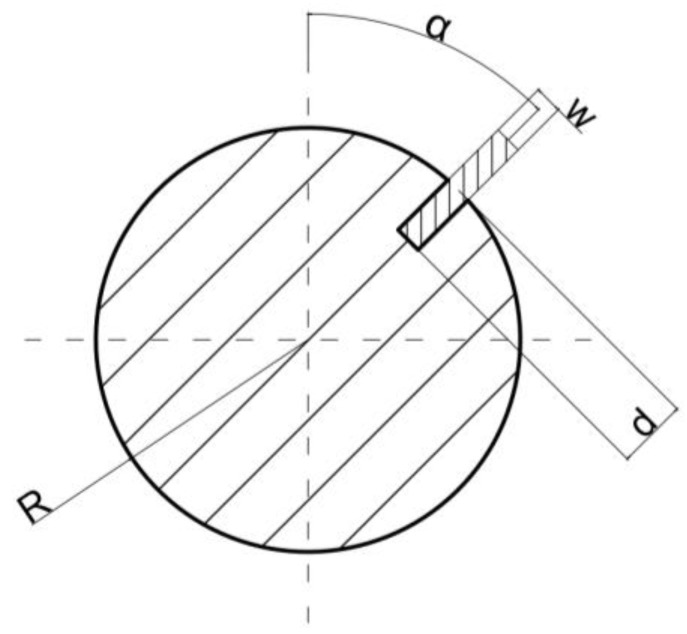
Utilized description of the tested object cross-section as a cylindrical object with a rectangular notch and marked parameters. R—object radius, d—depth of the notch, w—width of the notch, α—initial angular position of the notch with respect to the coil axes.

The object model may be described either by the distribution of the material in the cross-section of the sample [21] or a cylindrical model with a substitute defect [22]. In this paper, we utilize the second description, in which the object is fully described by four parameters, as presented in Figure 5.

The parameters of the model were updated as a result of the optimization algorithm. A new model was generated that provides input to the next iteration of FEM-based forward tomography transformation. The cycle continued until the optimization algorithm converged. Afterward, the tomography results were obtained as a parameter of the best-fit model with the smallest obtained value of the objective function (mean square difference between the normalized measurement and the modeling results).

A downhill simplex optimization method [23] was utilized as an optimization method for inverse tomography transformation. The sample’s electromagnetic parameters (magnetic permeability and electrical conductivity) were set to constant. Three parameters of the reference defect, as well as the sample radius (presented in Figure 5), were variables of the optimization algorithm.

To provide limits for the optimization algorithm, an external penalty function [24] was used. As a result of the algorithm operation, the value of any optimized variable may exceed acceptable limits. These limits are caused by the physical dimensions of the tomography setup and basic geometric dependencies (in order to maintain the rectangular shape of the reference defect). The parameter values were checked for whether they were within the limits before forward tomography transformation. The value of the penalty function is calculated from (2):

(2) $$x_{l}\leq x_{i}\leq x_{h}\;\to S=S^{\prime },\;(x_{l}\geq x_{i})\to S=S^{\prime }+1000+1000\times {\left(x_{i}-x_{l}\right)}^{4},\;(x_{i}\geq x_{h})\to S=S^{\prime }+1000+1000\times {\left(x_{h}-x_{i}\right)}^{4}$$

where:

*x_i_*—value of the optimized parameter;

*x_l_, x_h_*—lower and upper limit for a given parameter;

*S*—value of the penalty function;

*S′*—value of the penalty function obtained by checking the previous parameter.

The penalty function determines whether the value of the checked parameter is in the range of the set limits. If so, the value of the penalty function is 0. If the parameter is outside the limits, the value of the penalty function increases by a constant value and by the fourth power of the difference between the parameter value and the appropriate limit. The constant value is added to the penalty function in order to obtain a big enough value (much bigger than the values of the quality factor calculated for the normal parameters). The element connected with the fourth power of difference is added in order to provide the searching direction for the algorithm. Values furthest from the limits will return a higher value of the penalty function.

The parameters are checked in sequence. The value of the previous penalty function is added to the value of the currently calculated function. Thus, even a single parameter outside the limits will result in a non-zero value of the penalty function.

If the values of each checked parameter are inside the limits (*S* = 0 at the end of the verification), the parameters are used to create the model utilized in forward tomography transformation. Afterward, the value of the objective function is calculated as a mean square difference between the normalized measurement data and forward tomography transformation results. If even a single parameter is outside the corresponding limit, forward tomography transformation is not conducted. Instead, the value of the penalty function is returned to the optimization algorithm as an objective function.

The limits of the optimized parameters are presented in Table 1. The upper limit of radius *R* is caused by the physical distance between the excitation and measuring coil. A wider object simply will not fit into the measurement setup. The upper limit of the defect depth *d* is set to object radius *R*. The upper limit of defect width *w* is set to the value *w_max_*, which results from basic geometric dependencies in order to maintain the rectangular shape of the defect model.

(3) $$w_{max}=2\times \sqrt{2\times R\times d-d^{2}}$$

The lower limits of the parameters were selected empirically and were the smallest values that result in non-degenerated meshes for FEM-based forward tomography transformation.

### 2.4. Method of Forward Tomography Transformation

Inverse tomography transformation requires multiple applications of forward tomography transformation. This is a numerical reconstruction of the measurement process. Based on the given model of the object, the results are obtained, corresponding to the results of tomographic measurements for such an object. Forward tomography transformation is a crucial tool for proper inverse tomography transformation in eddy current tomography.

**Figure 6:**
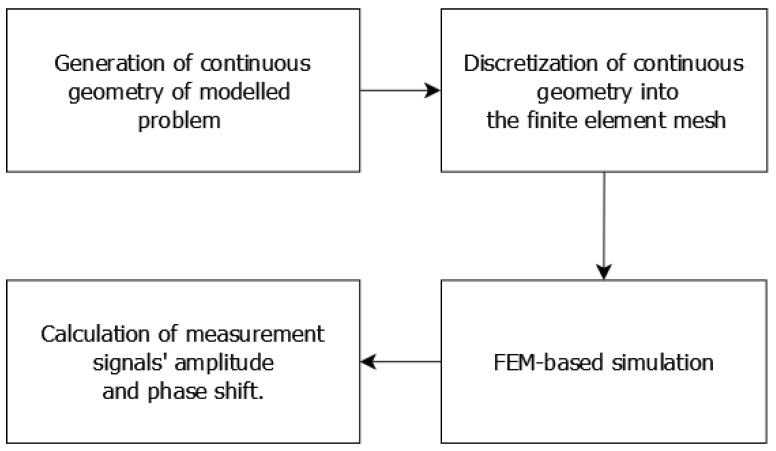
General scheme of FEM modeling for forward tomography transformation in eddy current tomography.

The modeling procedure in forward tomography transformation is a standard, FEM-based method [25], which follows the diagram presented in Figure 6.

Firstly, the continuous geometry of the analyzed problem must be prepared. Because the geometry changes between the simulations, due to changes in the tested object model, according to the optimization algorithm, this procedure was automated by developing the control script for creating files with a continuous geometry description of the given parameters. This was achieved in the Octave programming environment, which is an open-source alternative for MATLAB software. Scripts in Octave are responsible for data acquisition, optimization procedures, penalty function calculation, and control of multi-core FEM modeling.

**Figure 7:**
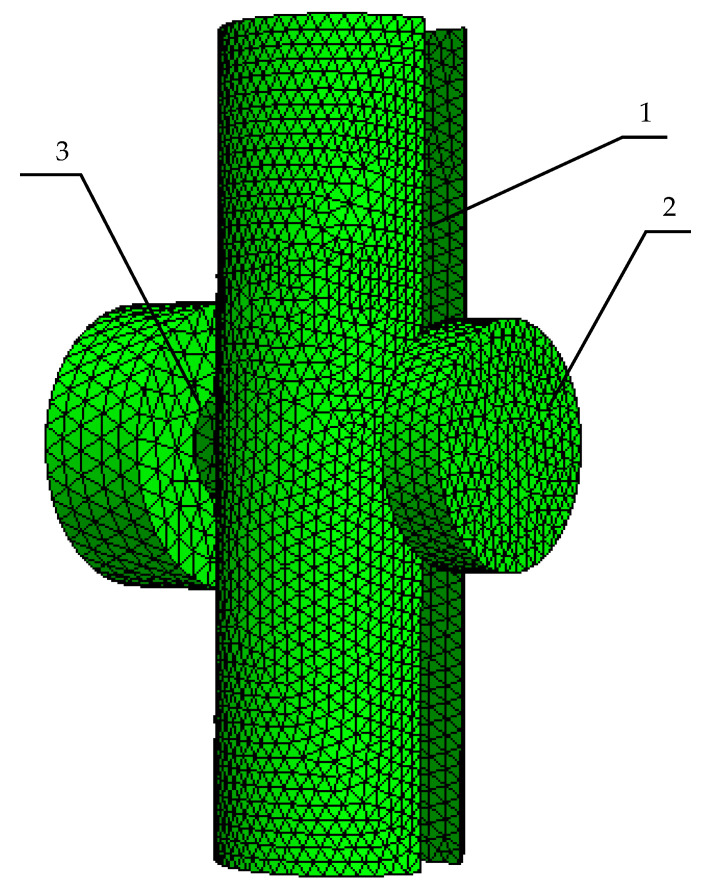
Exemplary finite element mesh for FEM-based forward tomography transformation in eddy current tomography. 1—model of the tested object, 2—model of the measuring coil, 3—model of the excitation coil.

The next step of forward tomography transformation is the discretization of the continuous geometry into the finite element mesh. This is achieved by Netgen software, which utilizes the Delaunay triangulation algorithm for improving the finite element quality. An exemplary view of the simplified eddy current tomography setup is presented in Figure 7. The measurement setup was limited to 4 objects: tested element, excitation and measuring coils, and an external air sphere, which provides the elements for calculating the magnetic field distribution in the entire tomography setup. The function of mechanical elements eliminated from the tomography setup (linear and angular actuators) is replaced by a proper description of the model’s continuous geometry, which simply places the object model in the desired linear and angular positions.

The finite element mesh is an input for the FEM solver. Elmer (v.9.0, CSC—IT Center for Science, Helsinki, Finland) [26], an open-source FEM solver, was chosen and validated on real [27,28] and theoretical [29] examples of magneto-dynamics simulation. The magneto-dynamics solver utilized in Elmer software solves the Maxwell equation in $\vec{A}$-V formulation, where $\vec{A}$ is a vector of magnetic potential, and V is the scalar electric potential. Equations (4) and (5) represent a weak formulation of Maxwell’s equation in the $\vec{A}$-V formulation:

(4) $$\overset{}{\underset{\Omega }{\int }}\sigma \frac{\delta \vec{A}}{\delta t}\cdot \nabla \upsilon d\Omega +\overset{}{\underset{\Omega }{\int }}\sigma \nabla V\cdot \nabla \upsilon d\Omega =\overset{}{\underset{\Omega }{\int }}\nabla \cdot \left(\sigma \vec{E}\right)\upsilon d\Omega -\overset{}{\underset{\delta \Omega }{\int }}\left(\sigma \vec{E}\right)\cdot \vec{n}\upsilon \mathrm{dS}\;=-\overset{}{\underset{\Omega }{\int }}\nabla \cdot \vec{g}\upsilon d\Omega -\overset{}{\underset{\delta \Omega }{\int }}\left(\sigma \vec{E}\right)\cdot \vec{n}\upsilon \mathrm{dS}$$

(5) $$\overset{}{\underset{\Omega }{\int }}\sigma \frac{\delta \vec{A}}{\delta t}\cdot \vec{\eta }d\Omega +\overset{}{\underset{\Omega }{\int }}\sigma \nabla V\cdot \vec{\eta }d\Omega +\overset{}{\underset{\Omega }{\int }}\frac{1}{\mu }\left(\nabla \times \vec{A}\right)\cdot \left(\nabla \times \vec{\eta }\right)d\Omega \;\;\;\;\;\;\;\;+\overset{}{\underset{\delta \Omega }{\int }}\left(\frac{1}{\mu }\nabla \times \vec{A}\right)\cdot \left(\vec{\eta }\times \vec{n}\right)\mathrm{dS}=\overset{}{\underset{\Omega }{\int }}\vec{g}\cdot \vec{\eta }d\Omega$$

Based on user-defined body forces, material parameters, and boundary conditions, the values of vector $\vec{A}$ and potential V are calculated in each element of the finite element mesh. These calculations are conducted with the linear optimization algorithm based on the bi-conjugate gradient-stabilized method [30].

During forward tomography transformation, body force was an alternating unit current driving the excitation coil. We used a single Dirichlet boundary condition. The magnetic potential was set to 0 on the surface of the external air sphere, which surrounded the model of the tomography setup. The diameter of this sphere was significantly bigger than any of the dimensions of the model of the tomography setup in order to obtain reliable values of the magnetic flux distribution [31].

The size of the mesh was selected based on the minimization of numerical noise and also considering the computational time. The results of single forward tomography transformation were filtered with the low-pass Butterworth filter with a cut-off frequency of 0.25 Hz. The values of the mean square difference between the original and filtered data were calculated for different sizes of finite elements in the meshes. With the lowering of the maximum size of the element, the noise influence became negligible, but the computational cost increased exponentially. Based on the results, the maximum size of the mesh element was set to 2 mm, and the mean time to calculate a single measurement point was 1003 s. Further lowering of the size of the mesh resulted only in higher computational costs (up to 2500 s for a single measurement point) and provided no significant improvement in results quality.

The size of the element considered both coils and the model of the tested element. The maximum size of elements in the external air sphere was set to 20 mm in order to lower the number of finite elements in the external layers of the sphere and thus reduce computational costs.

The obtained values of $\vec{A}$-V in the finite elements were then converted to the magnetic flux and electric field in each node of the finite element mesh. On their basis, it is possible to calculate the values of eddy currents in electrically conducting objects (based on Ohm’s law), magnetic field strength, or amount of induced heat (based on the Joule–Lenz law).

As a result of the singular simulation, the spatial distribution of the magnetic flux density in the tomograph setup was obtained. The value of the magnetic flux density in each finite element is represented as a complex number. The next step of the reconstruction of the measurement procedure was the numerical integration of the magnetic flux in the volume of the measurement coil. This corresponds to the voltage induction in the coil. Thus, data of the real part and the imaginary part of the magnetic flux density *B_re_* and *B_im_* are obtained. On this basis, the values corresponding to measurement signal amplitude *A* and phase shift *P* can be obtained as follows:

(6) $$A=\sqrt{{\left(B_{re}\right)}^{2}+{\left(B_{im}\right)}^{2}}$$

(7) $$P=arcsin\left(\frac{B_{im}}{\sqrt{{\left(B_{re}\right)}^{2}+{\left(B_{im}\right)}^{2}}}\right)$$

where:

*B_re_*—real part of the magnetic flux density numerically integrated into the volume of the measurement coil;
*B_im_*—imaginary part of the magnetic flux density numerically integrated into the volume of the measurement coil.

**Figure 8:**
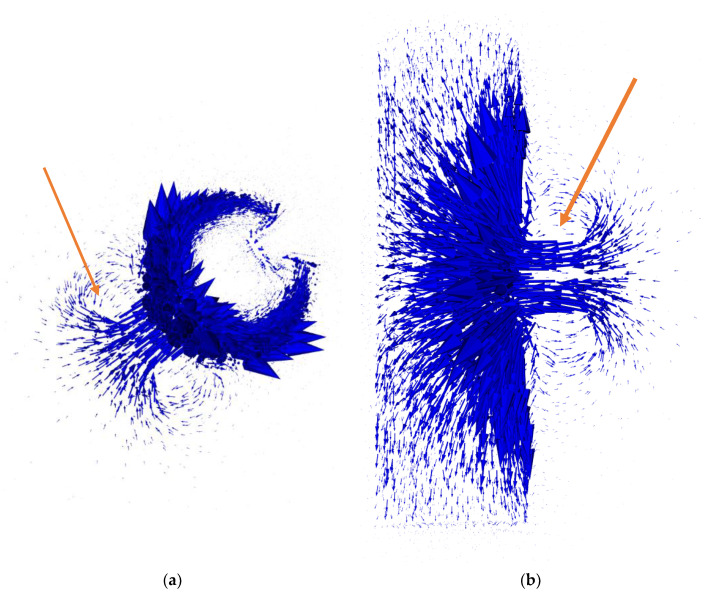
Exemplary spatial distribution of vectors of magnetic flux density in the tomography setup: (**a**) top view of the tested element; (**b**) side view. The arrow in each figure indicates the area of the excitation coil. All simulations were conducted in Elmer software. The length of an arrow represents the relative magnetic flux density in each node. The scale for absolute values is negligible, as all final results of tomography transformation are normalized to the 0–1 range.

**Figure 9:**
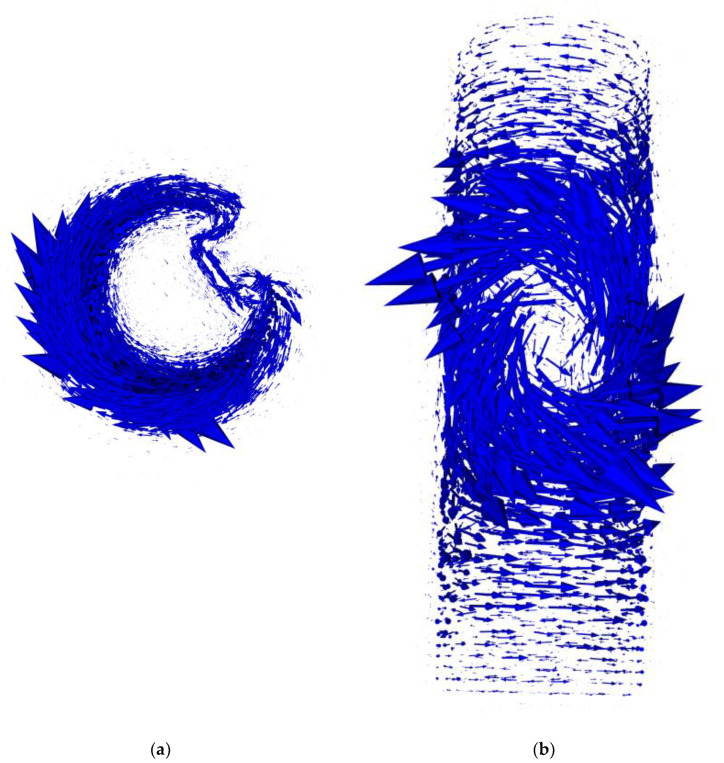
Exemplary distribution of vectors of induced eddy currents in the tomography setup: (**a**) top view of the tested element; (**b**) side view. All simulations were conducted in Elmer software. The length of the arrow represents the relative value of the induced current in each node. The scale for absolute values is negligible, as all final results of tomography transformation are normalized to the 0–1 range.

Exemplary results of FEM modeling for single measurement point in forward eddy current tomography transformation are presented in Figure 8 and Figure 9.

Visualization of magnetic flux and eddy currents distribution allows for a preliminary determination of the correctness of the applied FEM-based approach to forward eddy current transformation. Vectors of the magnetic flux density presented in Figure 8 have the highest density in the middle of the excitation coil (Area I, marked by arrows in Figure 8). The skin effect of the magnetic flux penetration is visible, as well as the fact that the lines of the magnetic flux are close to a closed contour.

Vectors of eddy currents presented in Figure 9 confirm the correctness of the application of three-dimensional FEM modeling. Eddy currents are induced in a plane parallel to the face plane of the excitation coil and their distribution and density are influenced by the shape of the tested object. The skin effect of eddy current induction is clearly visible. Figure 9a clearly shows that the skin depth of the eddy current does not influence the range of detection of the defect depth.

Forward tomography transformation requires multiple instances of FEM modeling for each measurement point, with corresponding linear and angular positions of the sample. Due to the disturbed axial symmetry of the sample, simulations were conducted, taking into account the rotation of the sample. The symmetry of the eddy current tomography setup [32] allowed limiting the number of measurement points in linear movement. The initial simulated measurement point in the forward eddy current tomography was 45 mm from the coil axis, whereas the final linear point was on the intersection of the sample axis and the coil axis.

Because inverse tomography transformation requires multiple instances of forward eddy current tomography transformation, any reduction in the computational cost of forward eddy current transformation multiplicatively reduced the computational cost of inverse eddy current tomography transformation. Forward tomography transformation was conducted only at selected points [33]. The point selection was an optimization task, where the objective function was the mean square difference between the original measurement data and data interpolated based on the selected points. As a result of the optimization, a smaller number of measurement points is selected, without a noticeable loss of information.

FEM-based forward tomography transformation requires full FEM modeling to be conducted for each of the selected measurement points. This requires all operations listed in the scheme presented in Figure 5: the generation of the continuous geometry of the problem, discretization of this geometry to a finite element mesh, FEM calculations, and data acquisition and aggregation. The whole procedure can be conducted on a single processor core. Additionally, simulations in different linear and angular positions are independent of each other, which allows for parallel computations. This significantly improves the computational cost of forward tomography transformation and, thus, inverse tomography transformation. The whole procedure is automated, executed, and controlled by scripts developed in the Octave programming environment.

**Figure 10:**
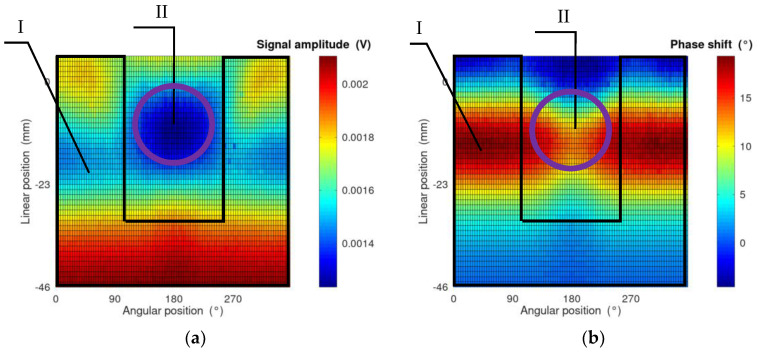
Exemplary results of forward tomography transformation for the sample with an 8 mm wide indentation. (**a**) Results of the signal amplitude as a function of the sample position; (**b**) results of the phase shift between the excitation and measured signals as a function of the sample position. Areas I and II represent projections containing information about sample radius and defect width, respectively.

An exemplary view of the results of forward tomography transformation for eddy current tomography is presented in Figure 10, conducted for a model of the tested cylindrical sample with a 30 mm diameter and a 120 mm height. Additionally, it contained a rectangular indentation with a depth of 13 mm and a width of 8 mm, similar to the real measured samples.

The high accordance of the obtained results with the measurement data is clearly visible. The differences are related to the value range of both the signal amplitude and its phase shift. The differences in signal amplitude are caused by the fact that the measuring coil was modeled as a single turn in contrast to the 100-turn coil in the measurement setup. Additionally, in the simulations, the excitation coil is powered by the unit current in contrast to the 2 A RMS AC current in the measurement setup. The values of the phase shift differ due to the influence of the signal conditioner, which consists of a signal amplifier and a band-pass filter. Thus, in order to compare the results of the measurement and FEM-based forward tomography transformation, all corresponding data had to be normalized into the 0–1 range.

## 3. Results

### 3.1. Inverse Tomography Transformation Results

**Figure 11:**
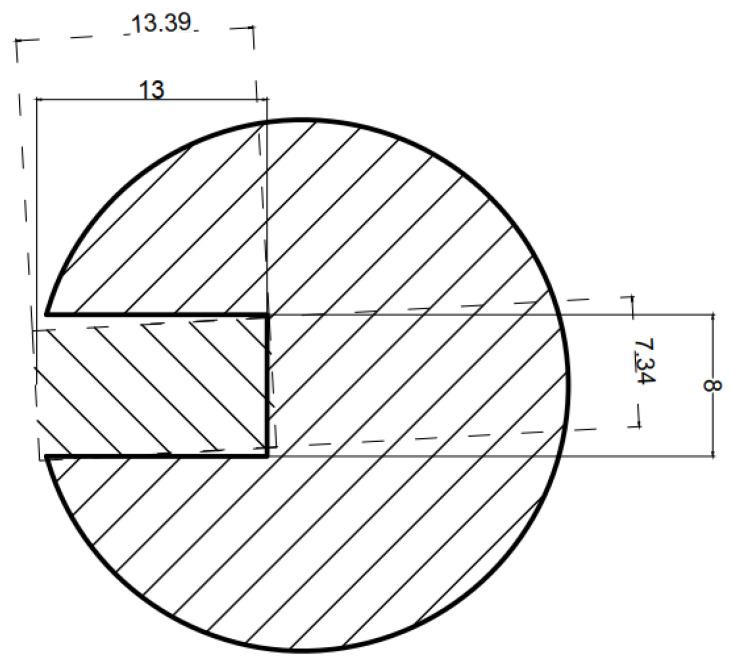
Exemplary comparison of real object parameters and parameters obtained by inverse tomography transformation for a sample with an 8 mm wide reference defect. Differences in object radius are not presented due to the legibility of the image.

As a result of the inverse eddy current tomography transformation, the values of the object parameters, which provide the highest accordance for the measurement data and FEM-based forward tomography transformation results, are obtained. The results, as well as the comparison with the real sample parameters, are presented in Table 2. An exemplary graphical comparison of the real discontinuities and obtained model defects is presented in Figure 11.

The reference defect parameters obtained by inverse tomography transformation provide satisfactory compliance with the real parameters. The inaccuracy of radius determination does not exceed 0.5 mm. The biggest difference for defect initial angular position is 3.2°, but other differences do not exceed 2°. The inaccuracy of defect width determination does not exceed 1 mm, and the defect depth was determined with a difference smaller than 2 mm when compared to the real sample parameters. This confirms the correctness of the proposed method, in which the parameters describing the object model are optimized with the continuous optimization algorithm.

### 3.2. Repeatability Analysis of the Results from Inverse Tomography Transformation

Repeatability analysis of the presented method was performed by conducting multiple iterations of inverse tomography transformation for the same measurement data. The sample with an 8 mm wide reference defect was measured on the eddy current tomography setup, and inverse tomography transformation was conducted three times. The obtained results are presented in Table 3. The mean value of the parameters obtained was calculated, as well as the standard deviation of the data series. Due to the small sample size (number of samples *n* < 30), the Student’s *t*-distribution was assigned to the results [34]. In order to determine the value of the expanded uncertainty of the measurement results, the value of the standard deviation was multiplied by the proper quantile of the Student’s *t*-distribution kν, α, where ν is the number of degrees of freedom, and α is the significance level. Assuming the confidence level *p* = 1 and α = 0.95, for the number of degrees of freedom ν = N − 1 (where N is the number of measurements in the series), the quantile value k_0.95, 2_ = 4.3 was obtained from the tables.

The presented results confirm that the proposed method of inverse tomography transformation for eddy current tomography is correct for the given objects. The obtained values of the sample parameters fit within the range of expanded uncertainty (for α = 0.05 and two degrees of freedom) when compared to the real parameters of the sample.

## 4. Discussion

The results of FEM-based forward eddy current tomography present high accordance with the measurement results. The noticeable difference in the range of amplitude signal changes is due to FEM model simplifications, the utilization of the unitary excitation current, and single-turn coil models. The offset difference between the results of the phase shift measurements and simulations is due to the presence of a signal conditioner in the eddy current tomography setup. This conditioner contains a band-pass filter that shifts the phase of the signal and an amplifier that increases the signal’s amplitude. Thus, in order to properly compare the modeling and measurements results during inverse tomography transformation, the data were normalized to the 0–1 range.

The inverse eddy current tomography transformation results presented in Table 2 clearly indicate that the utilized method is suitable for distinguishing the parameters of a defect in the sample. The results are obtained with high repeatability and are within the range of expanded uncertainty. It is worth emphasizing that such high accordance with the results is due to the similar shape of real defects and reference defects, the parameters of which were reconstructed in the optimization process. This emphasizes the need for the precise determination of the measurement aim and expected shape of the possible defects in the sample.

The results presented in Table 3 confirm the acceptable repeatability of the method. The deviation in the width of the reference defect (*w*) is significantly higher than the deviation of the other parameters. Information about other parameters is included in a larger number of projections during measurements. For example, information about the sample radius is included in Area I in Figure 10. On the other hand, information about defect width is mostly included in Area II, which corresponds to the situation where the defect is closest to the excitation coil. In other projections, the influence of eddy current disturbance on the defect is shielded by the sample itself.

This deviation can possibly be lowered by increasing the number of measurements in the region. This will require modification of the eddy current tomography setup in order to conduct measurement with variable angular steps (in contrast to a current fixed step measurement).

## 5. Conclusions

The methodology of inverse tomography transformation in eddy current tomography is presented. Due to the fact that induction of eddy currents is an ill-posed differential problem, the method utilizes FEM-based forward tomography transformation and optimization of the object model in the reconstruction procedure. The presented method utilizes continuous optimization of the parameters of a reference rectangular defect and may be suitable as an expansion of the classical eddy current method for non-destructive testing. This removes the need for an operator when conducting measurements and in the most crucial field of measurement data interpretation.

The obtained results of the reference defect are in high accordance with the parameters of the real defect, and the repeatability of the method is acceptable. The differences in multiple iterations of inverse tomography transformation are due to the numerical noise in FEM-based forward tomography transformation.

The measurement method is based on the standard eddy current non-destructive method. A comparison of the measurement results obtained on a flawless reference sample can provide general information about the occurrence of any defects in the test sample. For inverse transformation, this method requires an initial model with the number of cracks/defects assumed in advance. Thus, in the presented form, it cannot be used to determine the shape of the object. On the other hand, this method can be used in quality control to detect defects in manufactured elements. In potential industrial applications, previously computed patterns for the most common defects can be used. This will reduce the time to obtain information, as no FEM modeling will be required.

Further research will concern the application of different optimization algorithms. Tests will be conducted with the swarm particle optimization [35], the differential evolution algorithm [36] and the conjugate gradient method. The shape of the reference defect can be changed to be more universal or suitable for expected defects. Due to the fact that the number of parameters describing the reference defect significantly increases the computational cost, simple geometries should still be utilized. Possible alternatives for the shapes of reference defects are single cylinder, single sphere, or multiple spheres. Additionally, further research may include non-linear dependence of magnetic permeability as a function of external magnetic field [37,38] in modeling.

## Figures and Tables

**Table 1:** Limits of the optimized parameters of the model for inverse tomography transformation.

Parameter Symbol	Parameter Function	Parameter Lower Limit	Parameter Higher Limit
*R*	Object radius	5 mm	16 mm
α	Initial angle of defect	0°	360°
*d*	Depth of the defect	1 mm	*R*
*w*	Width of the defect	1.5 mm	*w_max_*

**Table 2:** Comparison of results of inverse eddy current transformation with real parameters of the sample.

Real Parameters of the Sample				Results of Inverse Tomography Transformation			
*R* (mm)	α (°)	*d* (mm)	*w* (mm)	*R* (mm)	α (°)	*d* (mm)	*w* (mm)
15.00	270	13.00	8.00	14.64	266.8	13.39	7.34
15.00	45	13.00	2.00	14.80	45.67	12.91	2.16
15.00	180	13.00	6.00	14.73	181.2	12.43	5.84
15.00	180	13.00	12.00	14.75	180	10.94	11.45

**Table 3:** Uncertainty analysis of inverse tomography transformation.

No.	Description	*R* (mm)	α (°)	*d* (mm)	*w* (mm)
1	Real parameters of the sample	15.00	270	13.00	8.00
2	Result 1	14.64	266.8	13.39	7.34
3	Result 2	14.81	268.3	13.21	7.84
4	Result 3	14.62	268.3	13.45	7.56
5	Mean value of the parameter	14.72	267.80	13.35	7.58
6	Standard deviation of the results	0.09	0.87	0.12	0.25
7	Expanded uncertainty	0.45	3.72	0.54	1.08
8	Difference between the real value of the parameter (Row 1) and the mean value of results obtained in three iterations of inverse tomography transformation (Row 5)	0.28	2.20	−0.35	0.42

## Data Availability

The data presented in this study are available on request from the corresponding author.
